# A biphasic model of lifespan in nematode *Caenorhabditis elegans* worm

**DOI:** 10.1098/rsos.220991

**Published:** 2023-02-01

**Authors:** Suhayl Mulla, Adele R. Ludlam, Aiman Elragig, Cathy Slack, Zita Balklava, Michael Stich, Alex Cheong

**Affiliations:** ^1^ Life and Health Sciences, Aston University, Birmingham B4 7ET, UK; ^2^ Engineering and Applied Science, Aston University, Birmingham B4 7ET, UK; ^3^ Faculty of Infectious and Tropical Diseases, London School of Hygiene and Tropical Medicine, London W1S 4BS, UK; ^4^ Departmento de Matemática Aplicada, Ciencia e Ingeniería de los Materiales y Tecnología Electrónica, Universidad Rey Juan Carlos, 28933 Móstoles, Spain

**Keywords:** lifespan curve, mathematical modelling, *Caenorhabditis elegans*, *Drosophila melanogaster*

## Abstract

Ageing research focuses on identifying lifespan modifiers and understanding and appropriately interpreting their effects. One of the most relevant quantities being studied is the shape of the survival curve that can reveal crucial information on the mechanism of action. Here, we introduce a bilogistic model to describe the shape of the lifespan curves of *Caenorhabditis elegans* populations. Using the corrected Akaike information criterion and the RMSE as goodness-of-fit tests, we show that the bilogistic model provides a better fit to the experimental data from nematode worms than other mathematical models and can identify and confirm biphasic lifespan data. Our parametric model offers a method to interpret replicate experiments data in terms of the shape parameters of the lifespan curve and enables robust statistical analysis of intra- and inter-group variance. We apply the model to novel lifespan data from *C. elegans* and *Drosophila melanogaster* and provide a rational statistical analysis of lifespan modifiers such as temperature and daf-16/FOXO mutation.

## 1. Introduction

Today, many societies face the challenge of a rapidly ageing population with associated health problems and increase in healthcare expenses. A crucial step towards our understanding of ageing is the identification and characterization of lifespan modifiers such as temperature [1], diet [2], pharmacological interventions [3] and genetic pathways [4]. The most common approach for testing a potential lifespan modifier is to use the non-parametric Kaplan–Meier estimator [5] followed by a log-rank test, which tests the null hypothesis that there is no statistically significant difference between the control and test groups [5]. This allows a qualitative comparison between a control and a test group, but not between replicates. Crucially, as it is only a significance test comparing two curves, it is unable to identify where differences occur or to provide an interpretation of them. One alternative is to perform parametric modelling of the data by fitting a mathematical function to the survival curve. Common distributions for lifespan data comprise simple exponential and sigmoidal distributions [6]. Two-parameter sigmoidal functions such as the Logistic [7], Weibull [8] or Gompertz [9] models use a death rate and a temporal quantification (e.g. median time lag, age-dependent mortality) as parameters to account for the shape and scale of the lifespan curve. Sigmoidal functions fit data points to a typical curve shape with a shoulder, a rapid decrease and a tail [6,10]. However, these models do not always adequately fit experimental data, leading to over-smoothing of the curves and unintentional discarding of features of the lifespan curve. For example, it has recently been shown for *Caenorhabditis elegans* that there are short-lived and long-lived worms resulting in survival curves which are different from a standard, monophasic decrease and that can be described as biphasic [11,12].

Here, we introduce a novel bilogistic model that describes and distinguishes two phases within the survival curve of a population, each with its own median survival. We apply this model to new experimental lifespan data for *C. elegans* obtained for different temperatures (worm lifespan depends on temperature [1,13]. The bilogistic model has four parameters, two median time constants, one death rate and the fraction of the population following either phase dynamics. We show that the bilogistic model allows for a clear identification of biphasic lifespan data for which it provides a significantly better fit of experimental data compared to several other models. This is demonstrated by the corrected Akaike information criterion (AICc) [14,15] that considers the different number of parameters of models, but we also apply other goodness-of-fit tests. We discuss a five-parameter extension (two different death rates), and a four-parameter variant (two different death rates but equal proportion between the subpopulations) of the model that can be appropriate to model specific experimental conditions.

## 2. Material and methods

### 2.1. *Caenorhabditis elegans* lifespan assay

The *C. elegans* strains N2 (wild-type) and *daf-16* (*mu86*) were acquired from the Caenorhabditis Genetics Center (CGC), University of Minnesota. All strains were maintained on solid nematode growth media (NGM) plates supplemented with nystatin (10 µg ml^−1^; Sigma) and seeded with *Escherichia coli* OP50 strain (CGC) at 20°C unless stated otherwise.

NGM plates used for lifespan assays were supplemented with 5-fluoro-2′-deoxyuridine (FUDR) (50 µM; Melford, UK) to prevent growth of unwanted offspring. Lifespan assays were conducted on a population of worms synchronized by alkaline hypochlorite treatment. Once synchronized, 50 (unless stated otherwise) L4 stage worms were transferred onto fresh NGM FUDR plates for the start of the experiment. Worms were transferred onto fresh plates weekly, but more frequently if the seeded OP50 was depleted or if in case of unwanted bacterial or fungal contamination. Day 0 is considered as the first day of adulthood and time points for events (death or censorship) were marked almost daily. Worms were classified into three categories at each time point: alive, dead or censored.

### 2.2. *Drosophila* lifespan assay

Control *w^Dah^* was derived by backcrossing *w^1118^* into the outbred wild-type *Dahomey* background. The *dfoxoΔ* flies were previously described [16]. Flies were raised and maintained on standard sugar/yeast medium [17]. Lifespan experiments were conducted at 25°C on a 12 : 12 h light/dark cycle at constant humidity. Flies were reared at standard density, allowed to mate for 24 h, sorted by sex and then transferred to experimental vials at a density of ten flies per vial. Approximately 100 flies were used per experimental replicate. Flies were transferred to fresh vials three times a week, and deaths were scored during transferral.

### 2.3. Survival curve plots

Kaplan–Meier survival curves were constructed by inputting events occurring at given time points into GraphPad Prism 9. Survival proportions at each time point were calculated and a log-rank (Mantel–Cox) test was conducted to statistically compare differences between two curves. *p-*values less than 0.05 were interpreted to show statistically significant differences between groups.

### 2.4. Lifespan models

In this study, we constructed a novel bilogistic model for fitting biphasic data, called Bilogistic 1kf (equation (2.1)). The *f* parameter splits the curve into two phases giving a weighting to each phase. There is a single death rate *k* and two time parameters *t*_1_ and *t*_2_, representing the median survival for each phase. We also constructed two variants of this model: Bilogistic 2kf with two different death rates instead of one (*k*_1_ and *k*_2_; equation (2.2)) and Bilogistic 2k, with two different death rates *k*_1_ and *k*_2_ but equal weighting of the two phases (setting *f* = 0.5; equation (2.3)). For each of the three models, the terms in the numerator ensure that at *t* = 0, the number of living animals at the start of the experiment is equal to *N*_0_, the known starting number, rescaled in each experiment to be 100%. The Bilogistic 2kf model with five parameters contains the other models (Bilogistic 1kf and Bilogistic 2k) as limit cases (*k*_1_ = *k*_2_ and *f* = 0.5, respectively) and can itself be interpreted as a generalization of the Whiting–Buchanan model with four parameters [18], where two different time constants are applied instead of one.

Bilogistic 1kf model:

2.1 $$N(t)=N_{0}\left[\;f\frac{1+e^{-kt_{1}}}{1+e^{k(t-t_{1})}}+(1-f)\frac{1+e^{-kt_{2}}}{1+e^{k(t-t_{2})}}\right].$$

Bilogistic 2kf model:

2.2 $$N(t)=N_{0}\left[\;f\frac{1+e^{-k_{1}t_{1}}}{1+e^{k_{1}(t-t_{1})}}+(1-f)\frac{1+e^{-k_{2}t_{2}}}{1+e^{k_{2}(t-t_{2})}}\right].$$

Bilogistic 2k model:

2.3 $$N(t)=0.5\;N_{0}\left[\frac{1+e^{-k_{1}t_{1}}}{1+e^{k_{1}(t-t_{1})}}+\frac{1+e^{-k_{2}t_{2}}}{1+e^{k_{2}(t-t_{2})}}\right].$$

The bilogistic models were compared against six models: the Whiting–Buchanan model with four parameters (equation (2.4) [18]), the Gompertz–Makeham model with three parameters (equation (2.5) [19]), the Logistic model with two parameters (equation (2.6) [7]), the Wilson model with two parameters (equation (2.7) [20]), the Weibull model with two parameters (equation (2.8) [8]) and the Gompertz model with two parameters (equation (2.9) [9]).

Whiting–Buchanan model:

2.4 $$N(t)=N_{0}\left[\;f\frac{1+e^{-k_{1}t_{\mathrm{lag}}}}{1+e^{k_{1}(t-t_{\mathrm{lag}})}}+(1-f)\frac{1+e^{-k_{2}t_{\mathrm{lag}}}}{1+e^{k_{2}(t-t_{\mathrm{lag}})}}\right].$$

Gompertz–Makeham model:

2.5 $$N(t)=N_{0}(e^{-ct-((a/b)(e^{\left(bt\right)}-1))}).$$

Logistic model:

2.6 $$N(t)=\frac{N_{0}}{1+e^{(t-t_{\mathrm{lag}})/k}}.$$

Wilson model:

2.7 $$N(t)=\frac{N_{0}}{1+{\left(t/t_{\mathrm{lag}}\right)}^{k}}.$$

Weibull model:

2.8 $$N(t)=N_{0}(e^{-{\left(at\right)}^{b}}).$$

Gompertz model:

2.9 $$N(t)=N_{0}(e^{-(a/b)(e^{\left(bt\right)}-1)}).$$

### 2.5. Statistical analysis

The experimental data have been analysed using various common statistical analysis packages that allow for minimum least-squares analysis, maximum-likelihood analysis, goodness-of-fit tests (including residual analysis/RMSE and AIC/AICc) and ANOVA tests.

The MATLAB (R2016a, v.9.0.0.370719, MathWorks) curve fitting toolbox was used to fit data using a nonlinear least-squares method (Trust-Region algorithm) to estimate parameters. The root mean square error (RMSE) was used for the goodness-of-fit statistical analysis of the different models using MATLAB. It represents the model performance and lower values of RMSE indicate smaller differences between actual data and fitted curve, and hence a better fit. To account for different numbers of parameters, the adjusted (or unbiased) RMSE is used:

$$\mathrm{RMSE}=\sqrt{\frac{\sum_{i=1}^{m}{\left(y_{i}-{\hat{y}}_{i}\right)}^{2}}{m-k},}$$

where *m* is the number of data points per experiment (replicate) of a lifespan curve, ${\hat{y}}_{i}$ is the predicted value at each data point and *k* is the number of parameters of the model. For a number *n* of replicates, the mean value and standard error of the mean (s.e.m.) were used. A customized residual analysis has also been used where several data points were binned (see Results). The statistical significance between two groups was examined using two-way analysis of variance (ANOVA) followed by Tukey multiple comparison tests using GraphPad Prism 9 (www.graphpad.com). Statistical significance at *p* < 0.05, *p* < 0.01, *p* < 0.001 and *p* < 0.0001 is indicated in the figures where appropriate.

The maximum-likelihood analysis to identify the optimal fit for each experiment was performed using the statistical programming language R (https://cran.r-project.org/index.html; v.3.5.1). Specifically, the R function mle2() was used, which relies on the optimization function optim() that by default uses a Nelder and Mead algorithm (https://www.rdocumentation.org/packages/bbmle/versions/1.0.20/topics/mle2). For Gompertz and Gompertz–Makeham models, we used a limited-memory modification of the BFGS quasi-Newton method. To account for different numbers of data points in each experiment and different numbers of parameters for each parametric model, the AICc was used to compare models. It is given by the equation

$$\mathrm{AICc}=\mathrm{AIC}+\;\frac{2k^{2}+2k}{m-k-1},$$

where *m* denotes the number of data points per experiment (replicate) and *k* denotes the number of parameters in the model and AIC = *2k − 2*ln(*L*) denotes the value obtained from the maximum-likelihood analysis, where *L* is the likelihood of a model with a certain set of parameters [14,15]. Lower values of AICc indicate a more reliable prediction.

Both methods, minimum least-squares and maximum likelihood, predict the parameter values for the model equations and allow us to fit a curve to the data. We specify the curve fitting method used in each figure.

## 3. Results

### 3.1. Variability in lifespan curves in *Caenorhabditis elegans*

**Figure 1.**
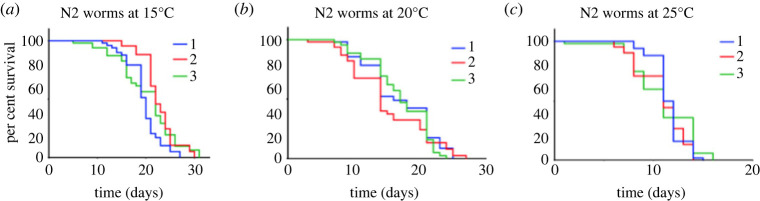
Experimental replicates of *C. elegans* lifespan. Representative experimental triplicate (coloured segments) of lifespan curves of N2 worms maintained at different temperatures ((*a*) 15°C, (*b*) 20°C, (*c*) 25°C) illustrates intra-group variability. Each experiment contained 100 worms.

The nematode worm *C. elegans* is a commonly used organism for studying lifespan. As previously described, lower temperatures generally result in longer lifespan in the worm [1]. Here, we use three experimental replicates to illustrate the variability in the lifespan curves for the same temperature, and the variability between three temperatures (15°C, 20°C and 25°C; figure 1*a–c*). Any single lifespan curve for 15°C, 20°C and 25°C is significantly different from any other lifespan curve at a different temperature when tested with the non-parametric Kaplan–Meier estimator followed by a log-rank test. For N2 worms, we have *n* = 20 replicates for 15°C, *n* = 15 replicates for 20°C and *n* = 14 replicates for 25°C. Therefore, the question arises of how to analyse quantitatively a set of replicates for identical experimental conditions and between different experimental conditions. For this, we use a parametric modelling approach.

### 3.2. Biphasic lifespan curves in *Caenorhabditis elegans*

**Figure 2.**
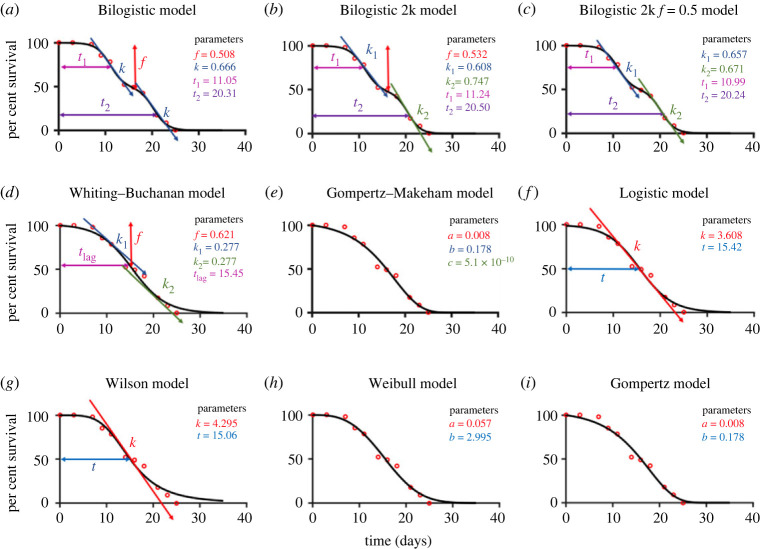
Curve fitting and parameter estimation of *C. elegans* lifespan. The Bilogistic 1kf model (*a*) and variants (*b*,*c*), together with common lifespan models (Whiting–Buchanan (*d*), Gompertz–Makeham (*e*), Logistic (*f*), Wilson (*g*), Weibull (*h*) and Gompertz (*i*)) were used to fit the same lifespan data of a single experiment (100 N2 worms maintained at 20°C). Data are represented by red circles and model fits (minimum least squares) by black curves. The parameters for each model are also given. An illustration of the meaning of the parameters is given for the Bilogistic models.

To address the questions raised above and using the observation that *C. elegans* populations can show a biphasic lifespan behaviour [11,12], we propose a bilogistic model (see Methods, equation (2.1)), named Bilogistic 1kf, for fitting the experimental data (least-squares fit or maximum likelihood, see Methods) and comparison against other models. In figure 2, we show the procedure for a single experiment (for 100 N2 worms at 20°C), where we performed a least-squares fit against Bilogistic 1kf and the other parametric models. The parameters of Bilogistic 1kf are as follows: the *f* value (which denotes the weighting or proportion of the two phases), the median survival times *t*_1_ and *t*_2_ and the death rate *k* for both phases (figure 2*a*). The phases represent a short-lived (1st phase) and a long-lived subpopulation (2nd phase). The Bilogistic 1kf model uses the same *k* value for both phases although we have also tested variants with two different *k* values (Bilogistic 2kf, equation (2.2); figure 2*b*) or with two different *k* values but equal phase distribution (Bilogistic 2k, equation (2.3); figure 2*c*). The Bilogistic 1kf model and its variants were used to fit the lifespan data points (figure 2*a*–*c*) and show a good visual agreement between data and curves. We fit the same data against a range of common lifespan models (see Methods, equations (2.4)–(2.9); figure 2*d*–*i*) and observe a systematic deviation for intermediate time moments (roughly, between Days 14 and 20) for all six models, and additionally for Days 3 and 7 for the Gompertz, Gompertz–Makeham and Logistic models. We quantify the goodness of fit systematically below, but already observe that the bilogistic models can fit the data well, clearly suggesting the existence of two phases, while there are systematic deviations for the other models which do not capture the biphasic nature of the survival curve.

### 3.3. Comparison of lifespan models using AICc and RMSE

To confirm or reject this observation and to compare the goodness of fit of the parametric models, we considered two independent measures to test for model selection: the AICc for the maximum-likelihood analysis and the RMSE for the minimum least-squares analysis.

**Figure 3.**
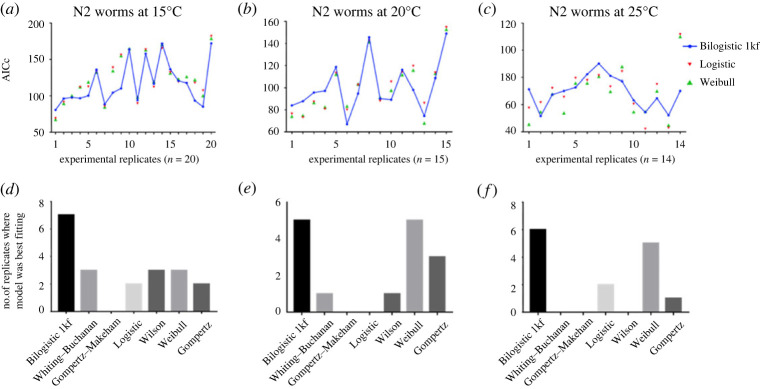
Analysis of lifespan models with the Akaike information criterion with small sample correction (AICc). The Bilogistic 1kf model and a panel of lifespan models were used to fit the lifespan data from N2 worms maintained at 15°C, 20°C and 25°C using the maximum-likelihood method. The AICc was calculated for each fit (*a*–*c*). The blue curve highlights the AICc values obtained from the Bilogistic 1kf model. Smaller values indicate better fits and the Bilogistic 1kf model was a best fit more often than the other models (*d*–*f*). The Weibull model was equally good for the 20°C data.

Since we are comparing models with different numbers of parameters, the AICc was used to account for the number of parameters in the model. A lower AICc score indicates best fit. From a set of experimental replicates of *C. elegans* maintained at 15°C (*n* = 20; figure 3*a*,*d*), 20°C (*n* = 15; figure 3*b*,*e*) and 25°C (*n* = 14; figure 3*c*,*f*), the Bilogistic 1kf model consistently scored a lower AICc than other models for all three temperatures. Only the Weibull model with two parameters was as good as our model for the 20°C dataset (figure 3*e*).

**Figure 4.**
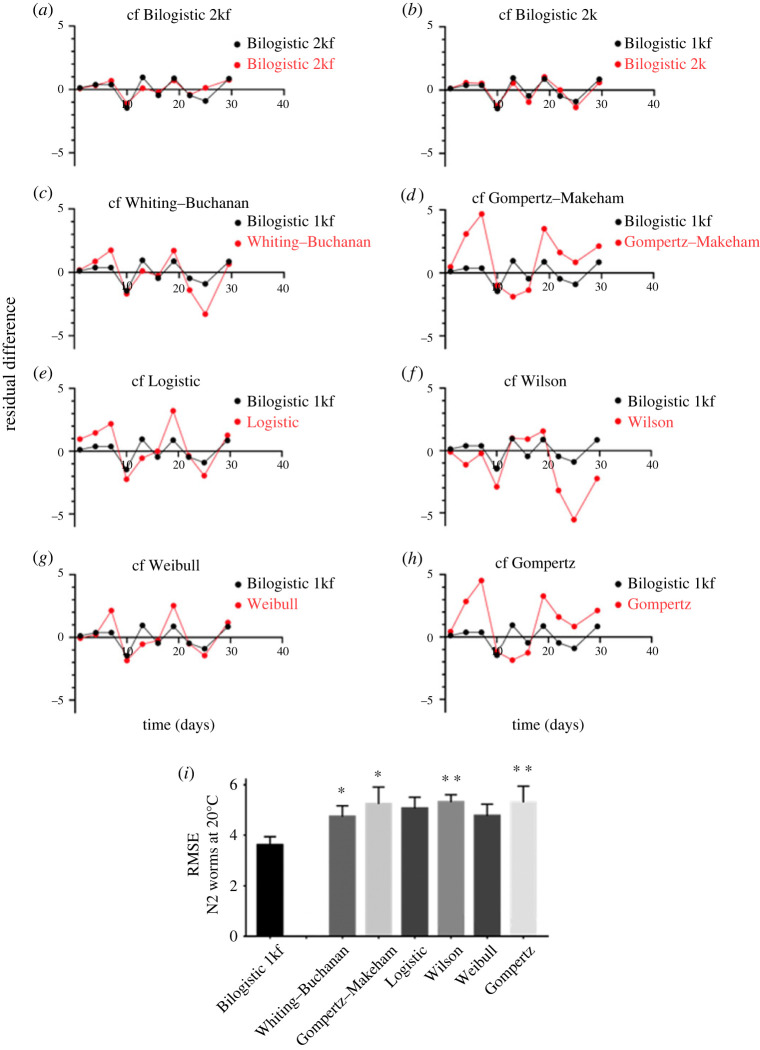
Analysis of lifespan models by residual difference analysis. (*a*–*h*) Comparison of residual differences from minimum least-square fits against the Bilogistic 1kf model (black) and against a panel of lifespan models (red) onto experimental replicate of N2 worms maintained at 20°C (*n* = 15). Binning was set to 3 days. The smaller the residual differences, the better the fit. (*i*) Analysis of the adjusted root mean square error (RMSE) for the panel of models (*n* = 15). Data are shown as mean ± s.e.m. *p < 0.05, **p < 0.01.

The adjusted RMSE is a measure of the residual differences between actual experimental lifespan data points and fitted model curve if models have a different number of parameters (see Methods). The residual differences are first calculated for each replicate of each dataset before undergoing a data binning procedure along the temporal axis to smooth the data. Residuals were grouped into bins of 2, 3 or 4 days and plotted (electronic supplementary material, figure S1). Plots which show the smallest residual error without systematic deviations throughout the lifespan indicate the best fit. The Bilogistic 1kf model shows the smallest residual error in all bins with a minimum of systematic deviations when compared to the other models (electronic supplementary material, figure S1), indicating that it is the most appropriate model for the data (we will comment on the use of the Bilogistic 2kf and Bilogistic 1k models in the Discussion). The results are relatively independent of the bin size, although bin size 2 performs slightly worse for the Bilogistic models. Following on, the adjusted RMSE was calculated for each curve. This type of analysis considers the varying number of parameters present in each model by giving a penalty for a model with more parameters. Using the data of the lifespan of *C. elegans* maintained at 20°C, we observed consistently smaller residuals using the Bilogistic 1kf model compared to other models (figure 4*a*–*h*). Consequently, we found significantly lower RMSE values for the Bilogistic 1kf when averaged over all replicates (figure 4*i*). We find similar results for worms maintained at 15°C and 25°C (electronic supplementary material, figures S2 and S3). Thus, the unbiased RMSE shows that the Bilogistic 1kf model is significantly better at fitting lifespan curves of *C. elegans* across different temperatures.

### 3.4. Analysis of the effect of temperature on nematode lifespan using the Bilogistic 1kf model

**Figure 5.**
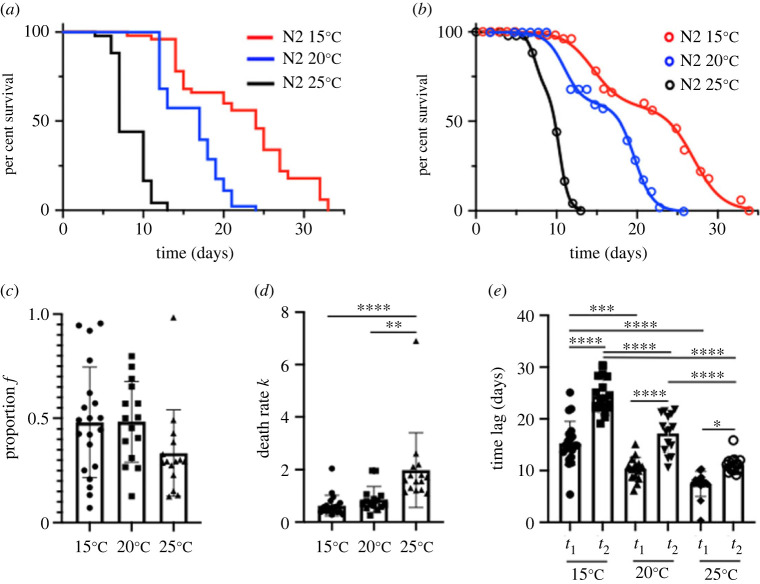
Effect of temperature on *C. elegans* lifespan. (*a*) Representative lifespan data for N2 worms maintained at 15°C, 20°C and 25°C and (*b*) fitted against the Bilogistic 1kf model with the maximum-likelihood method. (*c*–*e*) Analysis of the model parameters (*f*, *k*, *t*_1_ and *t*_2_) for lifespan curves of N2 worms maintained at 15°C (*n* = 20), 20°C (*n* = 15), 25°C (*n* = 14). Data are shown as mean ± s.e.m. *p < 0.05, **p < 0.01, ***p < 0.001 and ****p < 0.0001.

The lifespan curves for worms maintained at 15°C (*n* = 20 replicates), 20°C (*n* = 15 replicates) and 25°C (*n* = 14 replicates) were fitted against the Bilogistic 1kf model using the maximum-likelihood method (representative data in figure 5*a*,*b*) and the model parameters for each temperature condition were analysed. While at 25°C the *f* parameter is lower in many replicates compared to the parameters at 15°C and 20°C, this turned out not to be statistically significant since the overall spread is large (figure 5*c*). However, the death rate *k* is significantly increased as the temperature is increased from 15°C to 25°C or from 20°C to 25°C (figure 5*d*). The time parameters *t*_1_ and *t*_2_ were significantly different from each other, indicating the presence of two phases, although the difference becomes smaller and less significant for 25°C. Furthermore, both *t*_1_ and *t*_2_ were significantly decreasing as the temperature was increasing (figure 5*e*). Thus, the temperature has significant effects not only on the timing of the two phases, but also on the death rate.

### 3.5. Conserved effects of *daf-16/FOXO* deletion on lifespan

**Figure 6.**
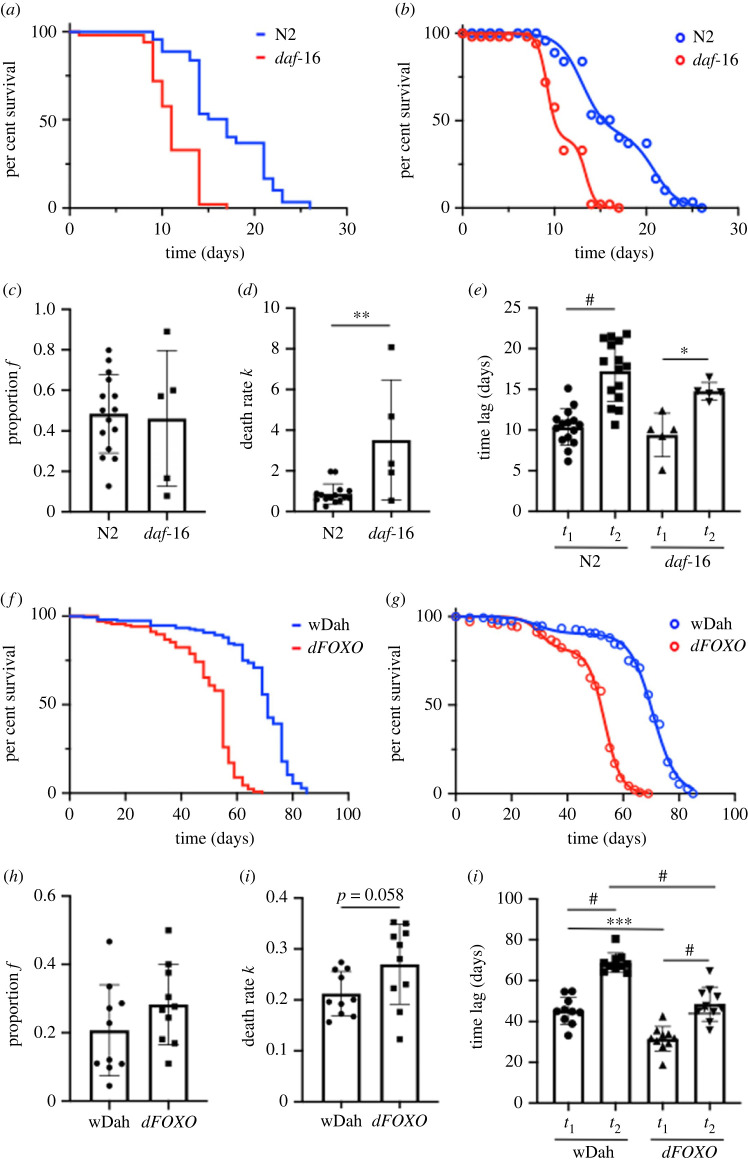
Effect of daf16/dFOXO mutation on lifespan. (*a*) Representative lifespan curves of N2 and daf-16 worms maintained at 20°C and (*b*) fitted against the Bilogistic 1kf model with the maximum-likelihood method. (*c*–*e*) Analysis of the model parameters (*f*, *k*, *t*_1_ and *t*_2_) for lifespan curves of N2 (*n* = 9) and daf-16 (*n* = 5) worms maintained at 20°C. (*f*) Representative lifespan curves of wild-type wDah and dFOXO mutant *Drosophila* flies and (*g*) fitted against the Bilogistic 1kf model with the maximum-likelihood method. (*h*–*j*) Analysis of the model parameters (*f*, *k*, *t*_1_ and *t*_2_) for lifespan curves of wDah (*n* = 9) and dFOXO (*n* = 5) flies. Data are shown as mean ± s.e.m. *p < 0.05, **p < 0.01, ***p < 0.001 and ****p < 0.0001.

We then asked whether the model could be applied to measure the effects of genetic manipulations on lifespan. We considered the Forkhead Box O (FOXO) nematode orthologue *daf-16*, known to play a critical role in regulating lifespan [4]. *daf-16* (*mu86*) worms lack daf-16*,* resulting in a significantly shorter lifespan (figure 6*a*), which could be fitted with our model (figure 6*b*). The loss of daf-16 did not affect the presence of the two phases and significantly increased the death rate *k* (figure 6*d*), with no effects on the other parameters.

We hypothesized that these effects of FOXO loss-of-function on lifespan are conserved in other organisms. To test this, we analysed the lifespans of *Drosophila melanogaster* lacking FOXO (*dfoxoΔ*), which also have a shorter lifespan than their control [16] (figure 6*f*) and which again could be better described by our model (figure 6*g*–*j*). The *Drosophila* lifespan curve has two phases as demonstrated by the two significantly different time parameters *t*_1_ and *t*_2_. However, the effects of *dfoxo* deletion in *Drosophila* did not exactly mirror our findings from the *daf-16*(*mu86*) worms*.* Thus, we observed a higher death rate in the mutant *dfoxoΔ* fly (*p* = 0.058) and statistically significantly decreased time parameters *t*_1_ and *t*_2_.

## 4. Discussion

Lifespan and survival are the results of a wide range of complex interactions within and among individual organisms and discerning the effects of lifespan modifiers is a challenging task. The log-rank test from the Kaplan–Meier estimator works as a basic statistical test [21], comparing two curves and assigning whether they are statistically significantly different. However, it does not provide any insights into the mechanisms of action of the lifespan modifier and, crucially, it cannot be used to test experimental replicates. Since the variability of lifespan experiments is high even for control groups, the problem lies in how to obtain a representative lifespan curve from multiple replicates.

Parametric analysis using e.g. Gompertz and Weibull models can address such questions [6]. By using only two parameters, these models propose that lifespan modifiers simply affect the monophasic gradient [22] or shift the lifespan curves along the time axis [23]. However, this assumes a monophasic lifespan with a uniform parametric distribution, which is not the case in many instances, and biphasic dynamics can be more pronounced depending on experimental conditions or animal strains [11,24]. Note that the asymmetric shape of the Gompertz and Gompertz–Makeham curves can be referred to as biphasic, but this does not capture the dynamics described here as we do not have an explicit exponential age-dependent component.

Biphasicness is a subtle effect and sometimes genetic, environmental or experimental parameters can mask or amplify it (e.g. see [25,26]). Our experimental data clearly displays more pronounced biphasic dynamics in certain conditions (e.g. *C. elegans* wild-type (N2 strain) at 20°C, *daf-16* (*mu86*)). When Vaupel [27] reviewed the different rates of mortality during the lifespans of insects and worms, a two-stage Gompertz model was normally found to be better at fitting the data. The question remains about what these two phases mean biologically. Vaupel [27] mused on the heterogeneity of the population where, although the individuals are genetically identical, they would differ from each other in terms of size, weight, robustness and frailty. More recently, analysis of the pharynx of *C. elegans* corpses revealed two distinct death types which could be separated as occurring at an early and late phase [12]. Similarly, use of the Smurf Assay, where gut permeability is assessed by a blue phenotype, in *Drosophila* flies, nematode worms and zebrafish also showed two phases of lifespan [28]. One recent paper uses the term ‘bimodal survival’ to describe the short-lived and long-lived cohorts of worm which could be adopting alternative physiological states due to unknown mechanisms [29].

Thus, the proposed bilogistic model attempts to capture the two phases, accounting for a proportion of the individuals dying earlier and the remainder dying significantly later as two distinct groups. The model can be interpreted as either an extension of a simple Logistic model (similar to the bilogistic model discussed by Meyer in a different context [30]) or as an extension of the Whiting–Buchanan model [18] with two lag phases instead of one.

Analysis of two goodness-of-fit tests, AICc for a maximum-likelihood model and adjusted RMSE for a least-squares model (for a recent discussion on these measures, see [31]), shows that the model Bilogistic 1kf fits the lifespan data on average better than other lifespan models (figures 3 and 4). Due to the high variability between replicates, the Bilogistic 1kf model is not always the most likely model, but it performs better than all other models tested. We emphasize that this conclusion does not depend on whether we use the AICc for a maximum-likelihood model or an adjusted RMSE for a least-squares model.

Adding an additional parameter such as a second death rate *k*_2_ or fixing the phase proportion *f* to 0.5 (while having two death rates *k*1 and *k*2) to modify the Bilogistic model to variants Bilogistic 2kf or Bilogistic 2k can significantly improve the fit or the advantage against other models, as shown in electronic supplementary material, figure S4. While we have used the Bilogistic 1kf model throughout this study, we observe that the Bilogistic 2kf model is more general and has a potentially wider applicability. It has five parameters, but simplifications can be applied in a straightforward way to obtain models with four parameters (Bilogistic 1kf, Bilogistic 2k) or three parameters (Bilogistic 1k) which may be appropriate for certain animal models or experimental conditions. We have, therefore, introduced a family of bilogistic models.

As is typical for survival models using logistic functions, the bilogistic models used here have not been derived from first principles but are justified *a posteriori* due to their predictive capacity. Our results agree with previous findings that logistic fittings provide better results than Gompertz–Makeham or Weibull fittings [32,33]. While a model with four or even five parameters has a higher risk of overfitting compared to a model with two or three parameters as for Weibull, Gompertz or Gompertz–Makeham, we emphasize that both goodness-of-fit tests have accounted for the numbers of parameters.

Comparing the parameters of the control curves to those for the test curves can reveal important clues to the action of the lifespan modifiers. In the case of high temperature, the death rate *k* increased while the phases shortened (*t*_1_ and *t*_2_). For the *daf-16* mutation, we only observed a statistically significant increase in the rate *k* but no differences in the other parameters. Interestingly, the equivalent mutation in *Drosophila* flies (dFOXO) instead showed a statistically insignificant increase in the rate *k* (*p* = 0.058) while both time parameters *t*_1_ and *t*_2_ were significantly reduced. These observations warrant further experimental investigations in mechanistic explanations and the effects of the DAF-16/FOXO mutations would suggest different mechanisms for the DAF-16/dFOXO mutations in both animals.

Although our experimental organisms are genetically homogeneous, lifespan analysis shows that subpopulations of short- and long-lived individuals exist. The present analysis does not reveal the mechanisms behind the observed biphasic dynamics which is beyond the scope of this article.

In conclusion, our study proposes a new parametric model for analysing lifespan assays. We show it is able to fit experimental data from the worms and flies, both of which exhibit biphasic dynamics. We have carefully considered the danger of overfitting our data and our residual difference analysis (valid for comparing models with the same number of parameters) and AICc and adjusted RMSE analysis (valid even for comparing models with different number of parameters, and therefore penalizing models with more parameters) show that the Bilogistic 1kf model offers a better fit than existing models. It can be easily inserted in commonly used statistical analysis programmes, and we hope that this paper encourages fellow scientists to apply parametric modelling to their lifespan data. Finally, analysis of the parameters from curve fitting enables comparison of lifespan assays both within similar groups and among different experimental conditions. Our model is, therefore, expected to facilitate the rational analysis of lifespan modifiers, e.g. through genetic analysis of long- versus short-lived subpopulations [12,34], and improve our understanding of their mechanisms of action.

Summarizing, the bilogistic model and its variants are variable enough to identify clearly biphasic survival data if present and to model the biphasic features of lifespan curves observed in response to environmental, genetic or other, such as pharmacological interventions.

## Data Availability

The datasets generated and analysed during the current study are included in the electronic supplementary material as Excel files [35].

## References

[1] Klass MR. 1977 Aging in nematode *Caenorhabditis elegans* - major biological and environmental-factors influencing life-span. Mech. Ageing Dev. **6**, 413-429. (10.1016/0047-6374(77)90043-4) 926867

[2] Colman RJ, Beasley TM, Kemnitz JW, Johnson SC, Weindruch R, Anderson RM. 2014 Caloric restriction reduces age-related and all-cause mortality in rhesus monkeys. Nat. Commun. **5**, 3557. (10.1038/ncomms4557) 24691430 PMC3988801

[3] Miller RA et al. 2014 Rapamycin-mediated lifespan increase in mice is dose and sex dependent and metabolically distinct from dietary restriction. Aging Cell **13**, 468-477. (10.1111/acel.12194) 24341993 PMC4032600

[4] Kenyon C, Chang J, Gensch E, Rudner A, Tabtiang R. 1993 A *C. elegans* mutant that lives twice as long as wild-type. Nature **366**, 461-464. (10.1038/366461a0) 8247153

[5] Goel MK, Khanna P, Kishore J. 2010 Understanding survival analysis: Kaplan-Meier estimate. Int. J. Ayurveda Res. **1**, 274-278. (10.4103/0974-7788.76794) 21455458 PMC3059453

[6] Lawless JF. 2014 Parametric models in survival analysis. In Wiley StatsRef: statistics reference online. Hoboken, NJ: John Wiley & Sons.

[7] Pletcher SD, Khazaeli AA, Curtsinger JW. 2000 Why do life spans differ? Partitioning mean longevity differences in terms of age-specific mortality parameters. J. Gerontol. A **55**, B381-B389. (10.1093/gerona/55.8.B381) 10952359

[8] Weibull W. 1951 A statistical distribution function of wide applicability. J. Appl. Mech. **18**, 293-297. (10.1115/1.4010337)

[9] Gompertz B. 1825 On the nature of the function expressive of the law of human mortality, and on a new mode of determining the value of life contingencies. Phil. Trans. R. Soc. **115**, 513-583. (10.1098/rstl.1825.0026) PMC4360127 25750242

[10] Xiong R, Xie G, Edmondson AE, Sheard MA. 1999 A mathematical model for bacterial inactivation. Int. J. Food Microbiol. **46**, 45-55. (10.1016/S0168-1605(98)00172-X) 10050684

[11] Zhang WB, Sinha DB, Pittman WE, Hvatum E, Stroustrup N, Pincus Z. 2016 Extended twilight among isogenic *C. elegans* causes a disproportionate scaling between lifespan and health. Cell Syst. **3**, 333-345.e334. (10.1016/j.cels.2016.09.003). 27720632 PMC5111811

[12] Zhao Y et al. 2017 Two forms of death in ageing *Caenorhabditis elegans*. Nat. Commun. **8**, 15458. (10.1038/ncomms15458) 28534519 PMC5457527

[13] Lee SJ, Kenyon C. 2009 Regulation of the longevity response to temperature by thermosensory neurons in *Caenorhabditis elegans*. Curr. Biol. **19**, 715-722. (10.1016/j.cub.2009.03.041) 19375320 PMC2868911

[14] Akaike H. 1973 Information theory and an extension of the maximum likelihood principle. In Second international symposium on information theory), pp. 267-281. Budapest, Hungary: Akademia Kiado.

[15] Sugiura N. 1978 Further analysts of the data by Akaike's information criterion and the finite corrections: further analysts of the data by Akaike's. Commun. Stat.-Theory Methods **7**, 13-26. (10.1080/03610927808827599)

[16] Slack C, Giannakou ME, Foley A, Goss M, Partridge L. 2011 dFOXO-independent effects of reduced insulin-like signaling in Drosophila. Aging Cell **10**, 735-748. (10.1111/j.1474-9726.2011.00707.x) 21443682 PMC3193374

[17] Bass TM, Grandison RC, Wong R, Martinez P, Partridge L, Piper MD. 2007 Optimization of dietary restriction protocols in Drosophila. J. Gerontol. Biol. Sci. Med. Sci. **62**, 1071-1081. (10.1093/gerona/62.10.1071) PMC4335187 17921418

[18] Whiting R, Buchanan R. 1992 Use of predictive microbial modeling in a HACCP program. In Proc. of the Second ASEPT Int. Conf.: Predictive Microbiology and HACCP, pp. 125-141. Cedex, France: ASEPT Laval.

[19] Makeham WM. 1860 On the law of mortality and the construction of annuity tables. Assur. Mag. J. Inst. Actuar. **8**, 301-310. (10.1017/S204616580000126X)

[20] Wilson DL. 1994 The analysis of survival (mortality) data - fitting Gompertz, Weibull, and logistic functions. Mech. Ageing Dev. **74**, 15-33. (10.1016/0047-6374(94)90095-7) 7934205

[21] Kaplan EL, Meier P. 1958 Nonparametric estimation from incomplete observations. J. Am. Stat. Assoc. **53**, 457-481. (10.2307/2281868)

[22] Vaupel JW, Johnson TE, Lithgow GJ. 1994 Rates of mortality in populations of *Caenorhabditis elegans*. Science **266**, 826. author reply 828. (10.1126/science.7973641) 7973641

[23] Stroustrup N, Anthony WE, Nash ZM, Gowda V, Gomez A, Lopez-Moyado IF, Apfeld J, Fontana W. 2016 The temporal scaling of *Caenorhabditis elegans* ageing. Nature **530**, 103-107. (10.1038/nature16550) 26814965 PMC4828198

[24] Lucanic M et al. 2017 Impact of genetic background and experimental reproducibility on identifying chemical compounds with robust longevity effects. Nat. Commun. **8**, 14256. (10.1038/ncomms14256) 28220799 PMC5321775

[25] Suda H, Sato K, Yanase S. 2012 Timing mechanism and effective activation energy concerned with aging and lifespan in the long-lived and thermosensory mutants of *Caenorhabditis elegans*. Mech. Ageing Dev. **133**, 600-610. (10.1016/j.mad.2012.07.007) 22898738

[26] Shoyama T, Shimizu Y, Suda H. 2009 Decline in oxygen consumption correlates with lifespan in long-lived and short-lived mutants of *Caenorhabditis elegans*. Exp. Gerontol. **44**, 784-791. (10.1016/j.exger.2009.09.006) 19808088

[27] Vaupel JW. 1997 Trajectories of mortality at advanced ages. In Between Zeus and the Salmon: the biodemography of longevity (eds K.W. Wachter, C.E. Finch), pp. 17-37. Washington, WA: DC: National Academies Press. 22973581

[28] Dambroise E, Monnier L, Ruisheng L, Aguilaniu H, Joly JS, Tricoire H, Rera M. 2016 Two phases of aging separated by the Smurf transition as a public path to death. Sci. Rep. **6**, 23523. (10.1038/srep23523) 27002861 PMC4802314

[29] Banse SA et al. 2019 Automated lifespan determination across *Caenorhabditis* strains and species reveals assay-specific effects of chemical interventions. Geroscience **41**, 945-960. (10.1007/s11357-019-00108-9) 31820364 PMC6925072

[30] Meyer P. 1994 Bi-logistic growth. Technol. Forecast. Soc. Change **47**, 89-102. (10.1016/0040-1625(94)90042-6)

[31] Portet S. 2020 A primer on model selection using the Akaike information criterion. Infect. Dis. Model. **5**, 111-128. (10.1016/j.idm.2019.12.010) 31956740 PMC6962709

[32] Vanfleteren JR, De Vreese A, Braeckman BP. 1998 Two-parameter logistic and Weibull equations provide better fits to survival data from isogenic populations of *Caenorhabditis elegans* in axenic culture than does the Gompertz model. J. Gerontol. Biol. Sci. Med. Sci. **53**, B393-B403. discussion B404-398. (10.1093/gerona/53A.6.B393) 9823735

[33] Yashin AY, Iachine IA, Begun AS. 2000 Mortality modeling: a review. Math. Popul. Stud. **8**, 305-332. (10.1080/08898480009525489)

[34] Tissenbaum HA. 2012 Genetics, life span, health span, and the aging process in *Caenorhabditis elegans*. J. Gerontol. A Biol. Sci. Med. Sci. **67**, 503-510. (10.1093/gerona/gls088) 22499764 PMC3410663

[35] Mulla S, Ludlam AR, Elragig A, Slack C, Balklava Z, Stich M, Cheong A. 2023 A biphasic model of lifespan in nematode *Caenorhabditis elegans* worm. Figshare. (10.6084/m9.figshare.c.6403443) PMC9890093 36756060

